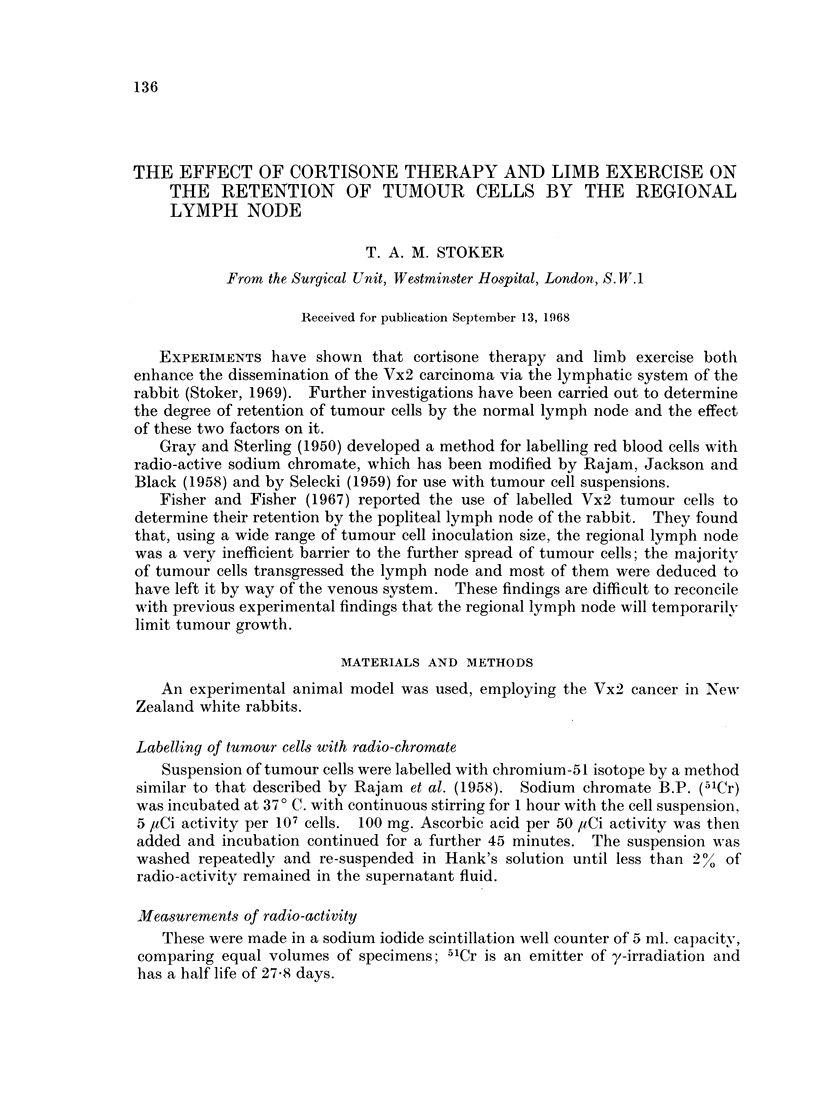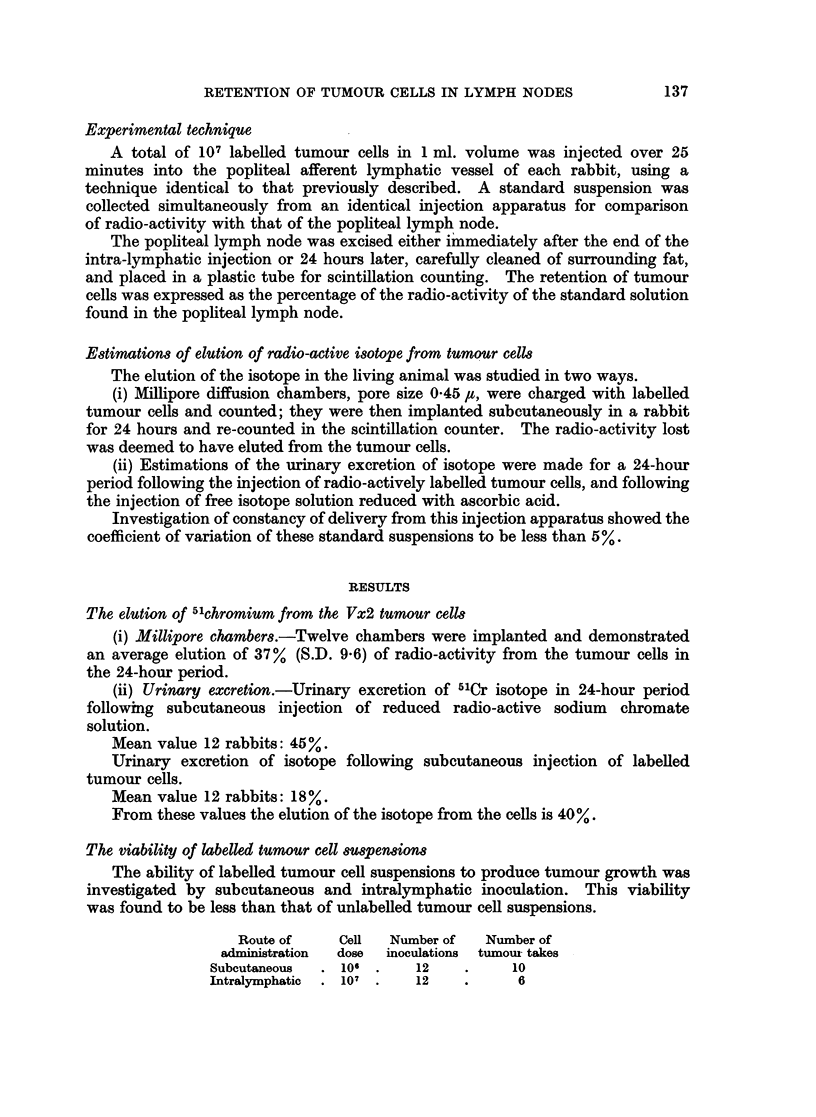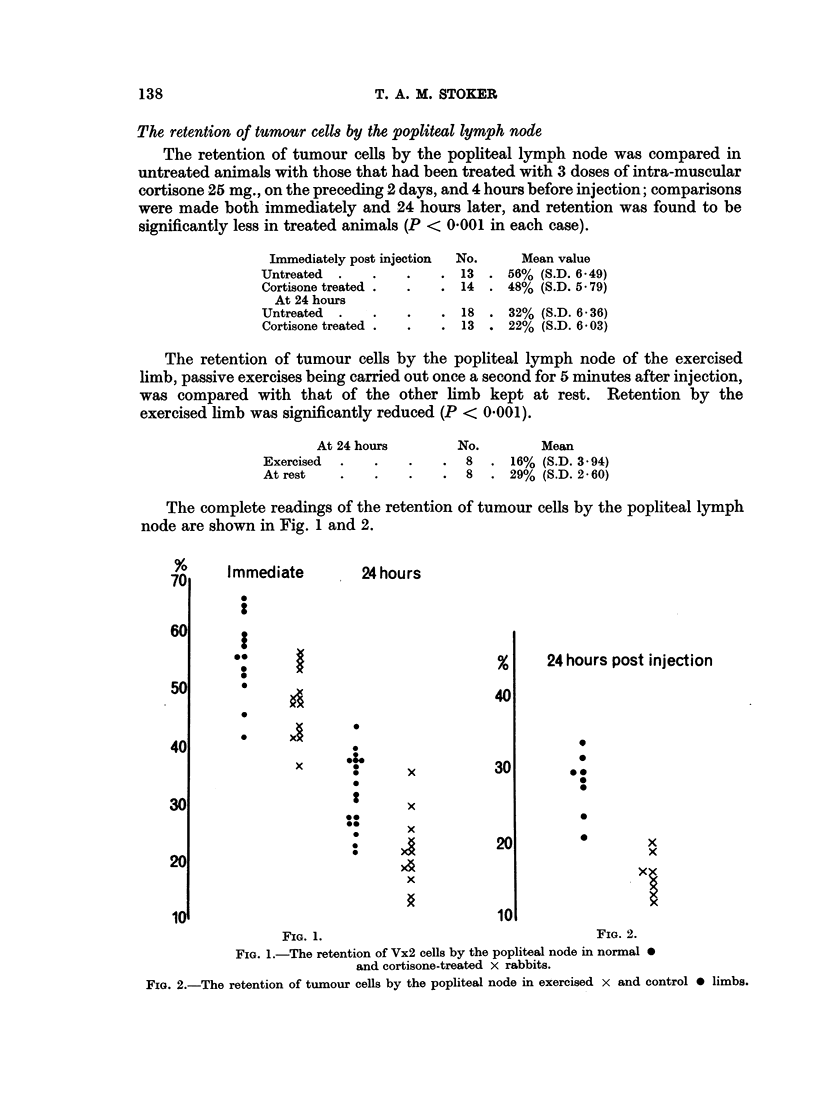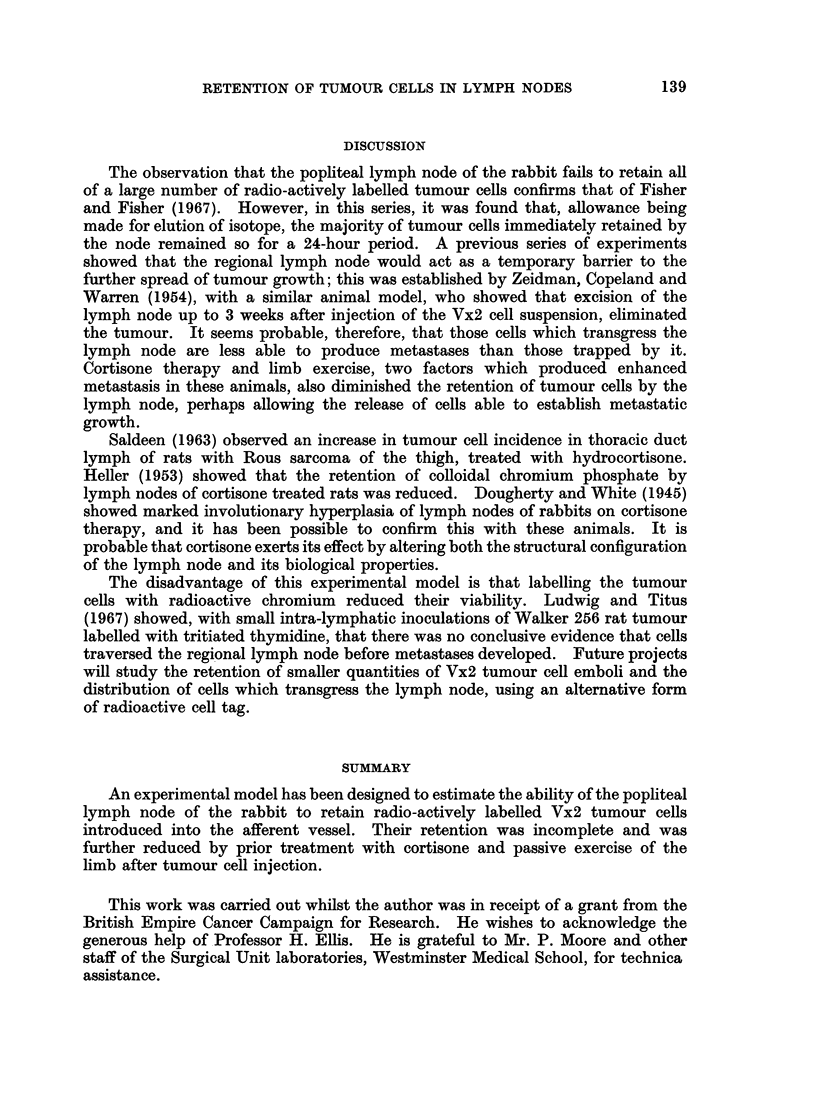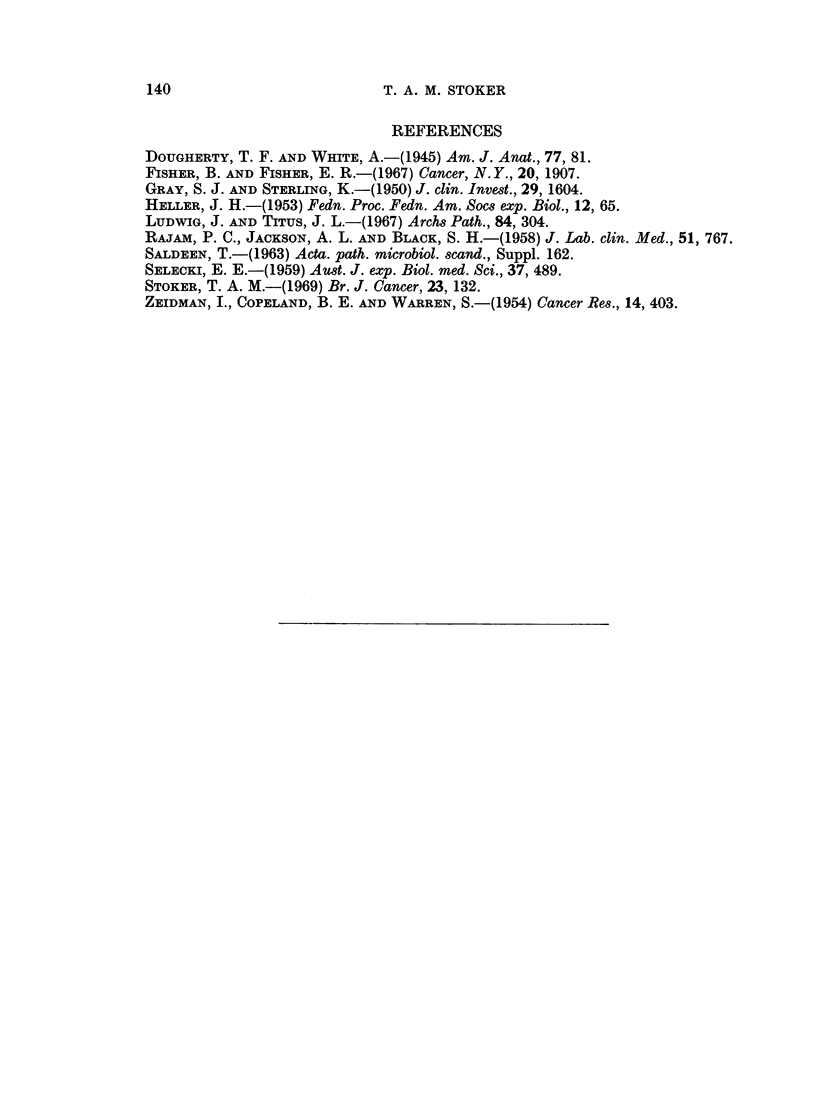# The effect of cortisone therapy and limb exercise on the retention of tumour cells by the regional lymph node.

**DOI:** 10.1038/bjc.1969.20

**Published:** 1969-03

**Authors:** T. A. Stoker


### THE EFFECT OF CORTISONE THERAPY AND LIMB EXERCISE ON THE RETENTION OF TUMOUR CELLS BY THE REGIONAL LYMPH NODE

T. A. M. STOKER

*From the Surgical Unit, Westminster Hospital, London, S.W.*1

Received for publication September 13, 1968

EXPERIMENTS have shown that cortisone therapy and limb exercise both enhance the dissemination of the Vx2 carcinoma via the lymphatic system of the rabbit (Stoker, 1969). Further investigations have been carried out to determine the degree of retention of tumour cells by the normal lymph node and the effect of these two factors on it.

Gray and Sterling (1950) developed a method for labelling red blood cells with radio-active sodium chromate, which has been modified by Rajam, Jackson and Black (1958) and by Selecki (1959) for use with tumour cell suspensions.

Fisher and Fisher (1967) reported the use of labelled Vx2 tumour cells to determine their retention by the popliteal lymph node of the rabbit. They found that, using a wide range of tumour cell inoculation size, the regional lymph node was a very inefficient barrier to the further spread of tumour cells; the majority of tumour cells transgressed the lymph node and most of them were deduced to have left it by way of the venous system. These findings are difficult to reconcile with previous experimental findings that the regional lymph node will temporarily limit tumour growth.

### MATERIALS AND METHODS

An experimental animal model was used, employing the Vx2 cancer in New Zealand white rabbits.

### *Labelling of tumour cells with radio-chromate*

Suspension of tumour cells were labelled with chromium-51 isotope by a method similar to that described by Rajam *et al.* (1958). Sodium chromate B.P. ($^{51}Cr$) was incubated at 37° C. with continuous stirring for 1 hour with the cell suspension, 5 $\mu$Ci activity per $10^7$ cells. 100 mg. Ascorbic acid per 50 $\mu$Ci activity was then added and incubation continued for a further 45 minutes. The suspension was washed repeatedly and re-suspended in Hank's solution until less than 2% of radio-activity remained in the supernatant fluid.

### *Measurements of radio-activity*

These were made in a sodium iodide scintillation well counter of 5 ml. capacity, comparing equal volumes of specimens; $^{51}Cr$ is an emitter of $\gamma$-irradiation and has a half life of 27·8 days.

*Experimental technique*

A total of $10^7$ labelled tumour cells in 1 ml. volume was injected over 25 minutes into the popliteal afferent lymphatic vessel of each rabbit, using a technique identical to that previously described. A standard suspension was collected simultaneously from an identical injection apparatus for comparison of radio-activity with that of the popliteal lymph node.

The popliteal lymph node was excised either immediately after the end of the intra-lymphatic injection or 24 hours later, carefully cleaned of surrounding fat, and placed in a plastic tube for scintillation counting. The retention of tumour cells was expressed as the percentage of the radio-activity of the standard solution found in the popliteal lymph node.

*Estimations of elution of radio-active isotope from tumour cells*

The elution of the isotope in the living animal was studied in two ways.

(i) Millipore diffusion chambers, pore size 0·45 $\mu$, were charged with labelled tumour cells and counted; they were then implanted subcutaneously in a rabbit for 24 hours and re-counted in the scintillation counter. The radio-activity lost was deemed to have eluted from the tumour cells.

(ii) Estimations of the urinary excretion of isotope were made for a 24-hour period following the injection of radio-actively labelled tumour cells, and following the injection of free isotope solution reduced with ascorbic acid.

Investigation of constancy of delivery from this injection apparatus showed the coefficient of variation of these standard suspensions to be less than 5%.

### RESULTS

*The elution of $^{51}$chromium from the Vx2 tumour cells*

(i) *Millipore chambers.*—Twelve chambers were implanted and demonstrated an average elution of 37% (S.D. 9·6) of radio-activity from the tumour cells in the 24-hour period.

(ii) *Urinary excretion.*—Urinary excretion of $^{51}$Cr isotope in 24-hour period following subcutaneous injection of reduced radio-active sodium chromate solution.

Mean value 12 rabbits: 45%.

Urinary excretion of isotope following subcutaneous injection of labelled tumour cells.

Mean value 12 rabbits: 18%.

From these values the elution of the isotope from the cells is 40%.

*The viability of labelled tumour cell suspensions*

The ability of labelled tumour cell suspensions to produce tumour growth was investigated by subcutaneous and intralymphatic inoculation. This viability was found to be less than that of unlabelled tumour cell suspensions.

| Route of administration | Cell dose | Number of inoculations | Number of tumour takes |
|---|---|---|---|
| Subcutaneous | $10^6$ | 12 | 10 |
| Intralymphatic | $10^7$ | 12 | 6 |

*The retention of tumour cells by the popliteal lymph node*

The retention of tumour cells by the popliteal lymph node was compared in untreated animals with those that had been treated with 3 doses of intra-muscular cortisone 25 mg., on the preceding 2 days, and 4 hours before injection; comparisons were made both immediately and 24 hours later, and retention was found to be significantly less in treated animals ($P < 0{\cdot}001$ in each case).

| Immediately post injection | No. | Mean value |
|---|---|---|
| Untreated . . . . | 13 | 56% (S.D. 6·49) |
| Cortisone treated . . . | 14 | 48% (S.D. 5·79) |
| At 24 hours | | |
| Untreated . . . . | 18 | 32% (S.D. 6·36) |
| Cortisone treated . . . | 13 | 22% (S.D. 6·03) |

The retention of tumour cells by the popliteal lymph node of the exercised limb, passive exercises being carried out once a second for 5 minutes after injection, was compared with that of the other limb kept at rest. Retention by the exercised limb was significantly reduced ($P < 0{\cdot}001$).

| At 24 hours | No. | Mean |
|---|---|---|
| Exercised . . . . | 8 | 16% (S.D. 3·94) |
| At rest . . . . | 8 | 29% (S.D. 2·60) |

The complete readings of the retention of tumour cells by the popliteal lymph node are shown in Fig. 1 and 2.

Fig. 1.

Fig. 2.

Fig. 1.—The retention of Vx2 cells by the popliteal node in normal ● and cortisone-treated × rabbits.

Fig. 2.—The retention of tumour cells by the popliteal node in exercised × and control ● limbs.

### DISCUSSION

The observation that the popliteal lymph node of the rabbit fails to retain all of a large number of radio-actively labelled tumour cells confirms that of Fisher and Fisher (1967). However, in this series, it was found that, allowance being made for elution of isotope, the majority of tumour cells immediately retained by the node remained so for a 24-hour period. A previous series of experiments showed that the regional lymph node would act as a temporary barrier to the further spread of tumour growth; this was established by Zeidman, Copeland and Warren (1954), with a similar animal model, who showed that excision of the lymph node up to 3 weeks after injection of the Vx2 cell suspension, eliminated the tumour. It seems probable, therefore, that those cells which transgress the lymph node are less able to produce metastases than those trapped by it. Cortisone therapy and limb exercise, two factors which produced enhanced metastasis in these animals, also diminished the retention of tumour cells by the lymph node, perhaps allowing the release of cells able to establish metastatic growth.

Saldeen (1963) observed an increase in tumour cell incidence in thoracic duct lymph of rats with Rous sarcoma of the thigh, treated with hydrocortisone. Heller (1953) showed that the retention of colloidal chromium phosphate by lymph nodes of cortisone treated rats was reduced. Dougherty and White (1945) showed marked involutionary hyperplasia of lymph nodes of rabbits on cortisone therapy, and it has been possible to confirm this with these animals. It is probable that cortisone exerts its effect by altering both the structural configuration of the lymph node and its biological properties.

The disadvantage of this experimental model is that labelling the tumour cells with radioactive chromium reduced their viability. Ludwig and Titus (1967) showed, with small intra-lymphatic inoculations of Walker 256 rat tumour labelled with tritiated thymidine, that there was no conclusive evidence that cells traversed the regional lymph node before metastases developed. Future projects will study the retention of smaller quantities of Vx2 tumour cell emboli and the distribution of cells which transgress the lymph node, using an alternative form of radioactive cell tag.

### SUMMARY

An experimental model has been designed to estimate the ability of the popliteal lymph node of the rabbit to retain radio-actively labelled Vx2 tumour cells introduced into the afferent vessel. Their retention was incomplete and was further reduced by prior treatment with cortisone and passive exercise of the limb after tumour cell injection.

This work was carried out whilst the author was in receipt of a grant from the British Empire Cancer Campaign for Research. He wishes to acknowledge the generous help of Professor H. Ellis. He is grateful to Mr. P. Moore and other staff of the Surgical Unit laboratories, Westminster Medical School, for technica assistance.

### REFERENCES

DOUGHERTY, T. F. AND WHITE, A.—(1945) *Am. J. Anat.*, **77**, 81.
FISHER, B. AND FISHER, E. R.—(1967) *Cancer, N.Y.*, **20**, 1907.
GRAY, S. J. AND STERLING, K.—(1950) *J. clin. Invest.*, **29**, 1604.
HELLER, J. H.—(1953) *Fedn. Proc. Fedn. Am. Socs exp. Biol.*, **12**, 65.
LUDWIG, J. AND TITUS, J. L.—(1967) *Archs Path.*, **84**, 304.
RAJAM, P. C., JACKSON, A. L. AND BLACK, S. H.—(1958) *J. Lab. clin. Med.*, **51**, 767.
SALDEEN, T.—(1963) *Acta. path. microbiol. scand.*, Suppl. 162.
SELECKI, E. E.—(1959) *Aust. J. exp. Biol. med. Sci.*, **37**, 489.
STOKER, T. A. M.—(1969) *Br. J. Cancer*, **23**, 132.
ZEIDMAN, I., COPELAND, B. E. AND WARREN, S.—(1954) *Cancer Res.*, **14**, 403.